# Brain-inspired biomimetic robot control: a review

**DOI:** 10.3389/fnbot.2024.1395617

**Published:** 2024-08-19

**Authors:** Adrià Mompó Alepuz, Dimitrios Papageorgiou, Silvia Tolu

**Affiliations:** Department of Electrical and Photonics Engineering, Technical University of Denmark, Copenhagen, Denmark

**Keywords:** robotics, nonlinear, model-based, learning, bio-inspired, brain, control, spiking

## Abstract

Complex robotic systems, such as humanoid robot hands, soft robots, and walking robots, pose a challenging control problem due to their high dimensionality and heavy non-linearities. Conventional model-based feedback controllers demonstrate robustness and stability but struggle to cope with the escalating system design and tuning complexity accompanying larger dimensions. In contrast, data-driven methods such as artificial neural networks excel at representing high-dimensional data but lack robustness, generalization, and real-time adaptiveness. In response to these challenges, researchers are directing their focus to biological paradigms, drawing inspiration from the remarkable control capabilities inherent in the human body. This has motivated the exploration of new control methods aimed at closely emulating the motor functions of the brain given the current insights in neuroscience. Recent investigation into these *Brain-Inspired* control techniques have yielded promising results, notably in tasks involving trajectory tracking and robot locomotion. This paper presents a comprehensive review of the foremost trends in biomimetic brain-inspired control methods to tackle the intricacies associated with controlling complex robotic systems.

## 1 Introduction

The field of robotics is advancing toward increasingly sophisticated robots that are able to perform tasks previously reserved only for humans due to their high complexity, especially when performed in unstructured environments. Such tasks range from human-robot interaction with compliance and motion constraints, to handling and manipulating objects of arbitrary shapes and materials in a dexterous manner, to legged-robot navigation in challenging environments. In many of these tasks, the robots being developed feature new structural materials and ways of actuation, and often present a high number of degrees of freedom. These include anthropomorphic musculoskeletal robotic systems (Diamond et al., 2012; Asano et al., 2017), soft-robotic arms and grippers (Cianchetti et al., 2018; Walker et al., 2020) and other robots such as walking robots (Coelho et al., 2021; Lyashenko et al., 2021). Novel ways of actuation include artificial muscles (Carpi, 2016; Mirvakili and Hunter, 2018). The high dimensionality and non-linearities present in these systems as well as the increasing complexity of the tasks the robots need to perform, pose a challenging control problem.

Conventional model-based control approaches guarantee strong stability properties of the controlled system and prescribed accuracy, even in the presence of structured and unstructured uncertainties. However, their design complexity scales very poorly with dimensionality and, therefore are difficult to generalize, maintain and tune in connection to complex robot tasks. On the other hand, relying on model-free or learning-based solutions, such as machine learning and statistical modeling methods can efficiently manage extensive system dimensions. Yet, they come with heavy computationally demands, struggle with adaptability to different scenarios and lack assurance in stability and robustness.

**Figure 1:**
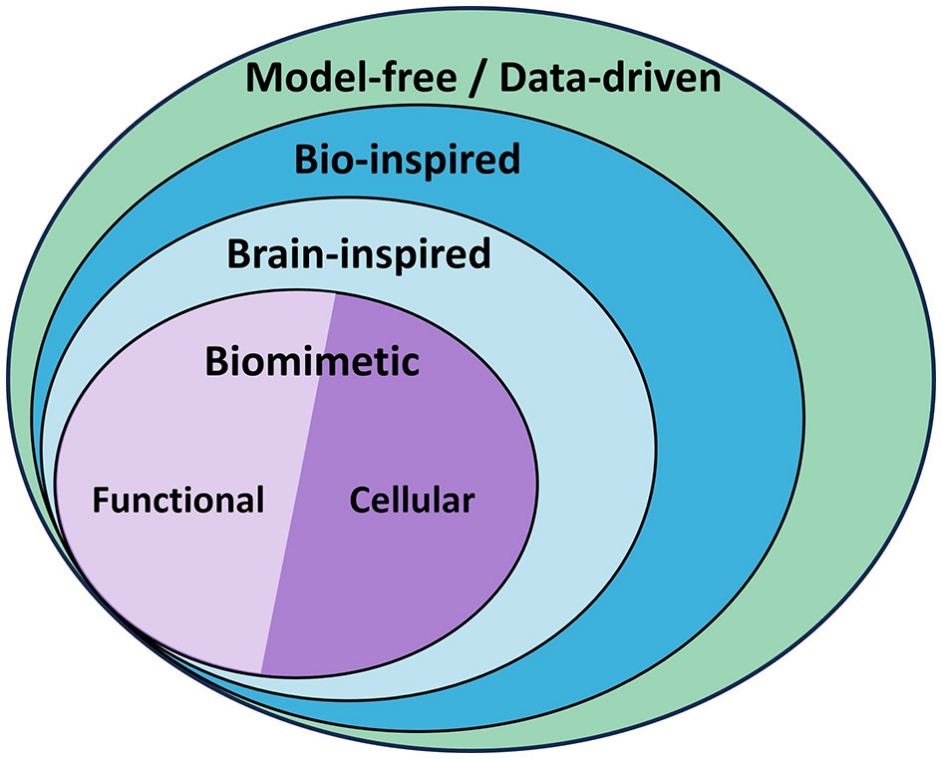
*Model-free* and *data-driven* controllers categorization. Model-free controllers may work without a model (Fliess and Join, 2013) or rely on data to obtain it. *Bio-inspired* control draws inspiration from biology to different degrees to design the controller, and *brain-inspired* control focuses specifically on methods inspired by the brain. When these methods closely mimic the processes and structures in the brain, they are referred to as *biomimetic* controllers, and they can just model the overall behavior with various function-approximation methods (*functional* approach), or the neuronal circuits and cells that give rise to such behavior (*cellular* approach). This review focuses on biomimetic control (BC).

Taking inspiration from biology, where humans and animals are able to gracefully and efficiently perform complex motion tasks, the scientific community of robotics has started pursuing research on new control strategies that are based on biological learning principles and architectures. More specifically, control schemes that reflect brain structures relevant to motor control have recently become central in pursuing efficient adaptive control of complex motion systems. These schemes are often referred to as *Brain-Inspired Control*. Brain-inspired control is included in the broader category of bio-inspired control, which accounts for any control method drawing inspiration from biology. Often bio-inspired control may only approximate biology vaguely and on a high abstraction level. The methods that more closely model the working principles of biological systems, in this case the brain, are referred to as biomimetic control (BC) methods. Figure 1 shows a diagram with this classification.

This study presents a review of *BC* methods applied to robotics. Specifically, it focuses on those methods that replicate function, structure or cellular-level processes from certain brain areas involved in motor control, and some of their interconnections. The remainder of the paper has the following structure: Section 2 provides an overview of conventional control strategies for robotics, classifying them according to their use of analytical vs. data-driven modeling approaches; Section 3 delves deeper into the core concepts of brain-inspired controllers; Section 4 presents the most relevant works on BC on recent decades and classifies them by the brain areas they model and the robotics tasks they address, and finally Section 5 closes with some remarks and possible future trends.

## 2 Overview of robot control methods

In general, the standard procedure for control systems design can be divided into two steps: model identification and controller design. For complex systems, the model identification phase is usually the most laborious one since it requires extensive knowledge of the equations describing the system. The controller design phase can vary in difficulty depending on the requirements of the control task and the level of non-linearity of the controlled system, which leads to the choice of simpler or more advanced controllers.

Depending on the level of use of analytical modeling vs. empirical data measurements to fit a model, controllers can be classified into several categories. We distinguish four main ones: model-based controllers, model-free or data-driven controllers and hybrid controllers. Model-based controllers follow the standard two-step approach and fully rely on analytical knowledge of the system; model-free or data-driven controllers may not use a model at all or rely on data to obtain it, and may also include the controller design phase in the data-driven modeling procedure; hybrid controllers use empirical data but impose some constraints on the model which are usually informed by physics or related to the control task. Biologically or bio-inspired controllers may be included in the data-driven category and in some cases in the hybrid controllers one.

Next, a brief overview of different existing methods for the presented controller categories is introduced, showing their strengths and weaknesses and motivating the research interest in bio-inspired controllers.

### 2.1 Model-based control

Conventional model-based controllers face several challenges when applied to novel robotics systems (difficulty in obtaining analytical model, hard to tune, poor scalability), however some solutions have been proposed since they can be useful given certain simplifying assumptions and for certain tasks and scenarios. For humanoid, musculoskeletal, and walking robots, part of the challenge is in scaling for the many degrees of freedom, which implies that conventional analytical inverse kinematics and dynamics modeling can be used given enough computing power. Qiao et al. (2023) presents an overview of several control strategies, including model-based ones, for controlling musculoskeletal robots. For hexapod walking robots, Coelho et al. (2021) presents several kinematics and dynamics-based methods for control. For quadruped and bipedal conventional control methods, refer to De Santos et al. (2006) and Westervelt et al. (2018). In the case of soft robotics, the physical equations of motion are not trivial to model since the robots deform in a continuous way across their length and use novel actuation methods with significant nonlinearities. Different approximations exist, modeling the dynamics and kinematics to different degrees, trading between accuracy and efficiency. Santina et al. (2023) shows the state of the art of model-based control for soft-robotic systems.

### 2.2 Model-free and data-driven control

Model-free controllers have been gaining traction in the field of robotics in recent years for removing the need for a tedious analytical modeling phase in complex systems. Some literature (Fliess and Join, 2013) refers to model-free control as the set of methods that do not use any model to perform control, although they may have adaptive gains. The methods that use a model obtained through data, are more correctly referred to as data-driven controllers. These data-derived models may encompass first principles (Fasel et al., 2021) or can be black-box input/output representations. In this paper we do not make a hard distinction between the different variations of model-free controllers and we will use this term to refer to all of the model-free types.

The most relevant data-driven methods used for control can be classified into two main families of algorithms: machine learning (ML) and statistical modeling. Statistical modeling introduces fundamental assumptions about the data distribution, enhancing interpretability but constraining the level of complexity that the obtained models can represent. Some statistical modeling techniques used in robotics include (Vijayakumar et al., 2005; Nguyen-Tuong et al., 2009), with applications in novel robotic systems such as soft robots (Tang et al., 2022).

Machine learning algorithms include, among other methods, supervised learning and reinforcement learning (RL), which are the two most commonly used approaches for learning in robotics. The work in Singh et al. (2022) presents a survey of RL methods for general robotics systems, while Wang et al. (2021) presents machine learning-based control methods for soft robotics. Supervised learning can be used to learn a dynamics model of the robot and environment which is then used by an adaptive control policy in a model-based RL setting (Polydoros and Nalpantidis, 2017; Zhang and Mo, 2021).

In recent years, research in robotics has predominantly focused on ML algorithms, particularly on artificial neural networks (ANNs). ANNs excel in handling complex data but demand large data volumes, posing cost challenges. Moreover, they struggle with quick online adaptation, suffering from issues like catastrophic forgetting and slow adjustment to changes in the robot or environment (Kirkpatrick et al., 2017). Some ML (Hoi et al., 2021) and statistical modeling techniques (Vijayakumar et al., 2005; Nguyen-Tuong et al., 2009) offer online learning capabilities, however they are intricate to fine-tune and less effective with higher task complexity. Overall, these data-driven methods lack formal guarantees in robustness and stability due to their black-box nature, impeding mathematical analysis and limiting extrapolation beyond training data.

### 2.3 Hybrid control

Often data-driven methods can benefit from partial knowledge of the system which can be used to assist or reduce the extent of the learning task. Alternatively, some model-based control architectures can use a model that is obtained or tuned through data-driven methods. This gives rise to hybrid control methods, which combine model-based and model-free techniques to complement each other's shortcomings.

A common modeling choice for systems with complex nonlinear dynamics is to approximate their behavior with a set of physics-informed nonlinear dynamics equations whose coefficients are obtained in a data-driven way. Afterwards, a nonlinear control system can be built around this model, taking advantage of the equations obtained. A popular modeling technique in this category is the Sparse Identification of Nonlinear Dynamics (SINDy) (Brunton et al., 2016). It uses sparse regression to identify the most relevant terms in a library of candidate nonlinear functions, resulting in a concise model that captures the essential dynamics of the system, making it popular for its interpretability and computational efficiency. Some examples of robotics applications of SINDy are found in Chen et al. (2021) for trajectory tracking and in Bhattacharya et al. (2020) for soft-robot modeling.

Other hybrid controller methods include data-driven model predictive control (Berberich et al., 2021), which has a broad application in robotic systems, and the work in Reinhart et al. (2017), which was used to control a soft-robot arm.

Overall, the area of hybrid controllers is a relatively unexplored line of research that holds promising results for enhancing interpretability of data-driven methods while retaining good stability and robustness properties from model-based approaches.

## 3 Brain-inspired control paradigms

In the domain of model-free controllers, a different approach emerges by delving deeper into biological paradigms. Animals, and particularly humans, are capable of performing advanced motion tasks dexterously, learning new behaviors efficiently, and adapting to new physical situations and changes in the environment. This is realized by highly evolved brains, and specifically by their sensorimotor brain areas. These areas address different functions in motion control and complement each other, presenting a connective structure and activity that can be studied and modeled. While neuroscience still holds many mysteries, our current understanding of certain brain regions provides ample inspiration for developing novel computational methods capable of emulating their functionalities. By closely mimicking their operational principles, it becomes possible to attain their desired attributes, including sparse and efficient computations, lifelong learning and online adaptation (DeWolf, 2021). This approach holds the promise of resolving many challenges inherent in prevalent data-driven algorithms like ANN.

Brain-inspired control algorithms vary in their approximation of biology, spanning a spectrum from replicating solely high-level processes or functions to simulating the intricacy of neurons and neural circuits found within motor areas. On the highest abstraction level, some methods that are based on different learning approaches can be included, such as iterative learning (Wang et al., 2009) and active inference (Pezzato et al., 2020). ANNs for control and RL methods are also brain-inspired, however they only represent a very coarse approximation of the brain structure (ANNs), or a high level behavioral process (RL). Control approaches using these learning methods are excluded from this review, with the exception of some RL cases which are framed in the context of a specific motor brain area (e.g., the basal ganglia—BG).

This review focuses on the works that attempt to closely replicate the processes and structures of the motor brain areas. The controllers based on these methods are also referred to as BCs. There are two main approaches to mimicking the brain motor areas: the functional and the cellular ones. This was a distinction introduced for cerebellar models (Luque et al., 2014), and we extend it to other brain areas.

The functional point of view identifies the substructures of each motor area and aims to replicate their behavior with generic conventional learning or function approximation methods. Then it establishes the relationships between these substructures with the proper connections to mimic the transfer of information taking place in the biological counterpart. This approach does not harness the full potential of biological networks since the function approximation methods present their own limitations. Generally, this approach can be used to validate neuroscientific hypotheses concerning high level processes, e.g., evaluating the role of specific motor areas as a whole or their relationships with other areas.

The cellular point of view, also referred to as bio-plausible in this review, seeks to replicate the lower-level mechanisms of the brain motor areas by modeling these down to individual cells and microstructures. Models of neurons create the basis for building the emulated brain circuits, which are implemented by establishing the required synaptic connections. The implementation of cellular-level models still represents an approximation of the whole biochemistry involved in the neuronal processes, but gives rise to a desired behavior which is useful for robotics control. Additionally, this approach allows for testing neuroscientific hypotheses with finer detail than the functional modeling approach (Tolu et al., 2023), addressing not only the role of the emulated areas but also of more specific low-scale groups of neurons and their connections.

To learn and represent functions and behaviors, the bio-plausible neuron models are connected in a similar way as traditional artificial neural networks, but with inhibitory and excitatory connections, and communicate via spike signals. This distinctive feature gives rise to what are known as spiking neural networks (SNNs) (Maass, 1997; Ghosh-Dastidar and Adeli, 2009). Due to the spiking nature of the neurons, these networks present several desirable characteristics also present in biological networks conforming animal brains:

- The spike-based communication between neurons is a highly efficient way to convey information through the network, and this becomes evident when implemented in energy-efficient neuromorphic hardware.
- The network is sparsely activated, which means that only a small subset of the neurons are active at a given time, thus reducing the energy consumption further.
- The dynamic behavior of the neurons makes SNN a great candidate to represent time-dependent data, such as dynamic models in robotics.

Several studies have explored biologically plausible learning methods for spiking neural networks (SNNs) using spike-timing-dependent plasticity (STDP) (Feldman, 2012). These include unsupervised STDP-based models with adaptive mechanisms (Dong et al., 2023), supervised learning methods combining STDP with synaptic scaling and intrinsic plasticity (Hao et al., 2020), and online-learning models for hardware implementation (Qiao et al., 2019). These works aim to bridge the gap between biologically plausible approaches and backpropagation-based methods while providing insights into how learning occurs in biological systems. Taherkhani et al. (2020) show an overview of biologically plausible learning methods for SNNs.

Some other studies have explored alternative approaches to make SNN training and deployment more efficient and robust, albeit not retaining complete biological plausibility. Tavanaei et al. (2019) provide an overview of methods for training deep SNNs, while Kim et al. (2023) analyze temporal information dynamics during training. Yao et al. (2023) introduce an attention module to improve performance and energy efficiency, and Yang et al. (2023a) propose a multi-scale learning rule with dendritic predictive characteristics. Yang and Chen (2023a,b), and Yang et al. (2023b) present information-theoretic learning approaches using nonlinear information bottleneck principles and explore the design space of the information bottleneck framework to improve robustness, accuracy, and power efficiency in SNNs.

For an overview of the uses and properties of SNNs as well as their training methods, refer to Yamazaki et al. (2022) and Pietrzak et al. (2023) respectively.

The cellular-level approach can become computationally expensive depending on the level of biological fidelity and the number of simulated neurons. This is problematic for current common computing hardware such as GPUs and CPUs, but neuromorphic hardware (Young et al., 2019; Rathi et al., 2023) addresses this issue by implementing the behavior of neurons on a physical level or *in-silico*. This substantially enhances computational power and efficiency. Some neuromorphic platforms that have been developed by semiconductor companies include Intel^®^Loihi (Davies et al., 2018) with programmable spiking neural network features, IBM^®^TrueNorth (Akopyan et al., 2015) with 1 million neurons and 256 million synapses. Others have been used for large-scale research projects such as the Human Brain Project (Amunts et al., 2016), including SpiNNaker (Furber et al., 2014) designed for large-scale spiking neural network modeling, and BrainScaleS (Pehle et al., 2022) combining analog spiking neural network emulation with digital components. Several works on robotics have used some of these platforms to different degrees, which will be presented later.

Other research groups have developed alternative neuromorphic platforms that leverage custom mixed-signal circuits and specialized digital architectures to emulate the behavior of biological neurons and synapses with varying levels of abstraction and realism. For example, Yang et al. (2022b) propose a hybrid neuromorphic platform integrating multiple granules of SNNs, demonstating the replication cognitive activities like motor learning and action selection. Yang et al. (2021) focus on a large-scale cerebellar network model and architecture for supervised motor learning, with over 3.5 million neurons, better mimicking the biological cerebellum's structure. Additionally, Yang et al. (2024a) presents a neuromorphic architecture with dendritic on-line learning (NADOL) for brain-inspired intelligence on embedded hardware, exhibiting superior learning capabilities compared to GPU platforms. Emphasizing on fault-tolerance, the works Yang et al. (2022a, 2024b) present neuromorphic frameworks capable of robust learning and decision-making. While Yang et al. (2022a) focuses on context-dependent learning of stimulus-response associations, Yang et al. (2024b) integrates visual perception with decision-making, demonstrating high accuracy and minimal latency.

Neuromorphic computing remains an emerging field, especially concerning large-scale simulations, yet it holds vast potential. As hardware progresses, these challenges are poised to diminish, offering immense promise for the future. Presently, implementations on conventional computing platforms lean toward simplified neuron models, retaining their fundamental characteristics while boosting performance and enabling greater scalability.

This paper predominantly reviews on cellular-level implementations within BCs, as they exhibit considerable promise in replicating brain functions in the future. This potential amplifies as our structural understanding of the brain evolves and neuromorphic hardware advances. However, it also touches upon significant work employing a functional approach, as these initiatives lay the groundwork for future advancements and possess adaptability for accommodating SNNs. The main works on BCs found in literature are presented in the next section.

## 4 Biomimetic control models

Over the past few decades, the accumulation of evidence concerning how the brain's motor areas function has inspired the creation of computational models. These models aim to mimic their behavior and utilize their characteristics in robotics applications. The upcoming sections detail the latest developments in BCs, striving to merge the exploration and validation of current neuroscientific theories with the practical task of controlling robots, encompassing both simulated environments and real-world scenarios.

**Figure 2:**
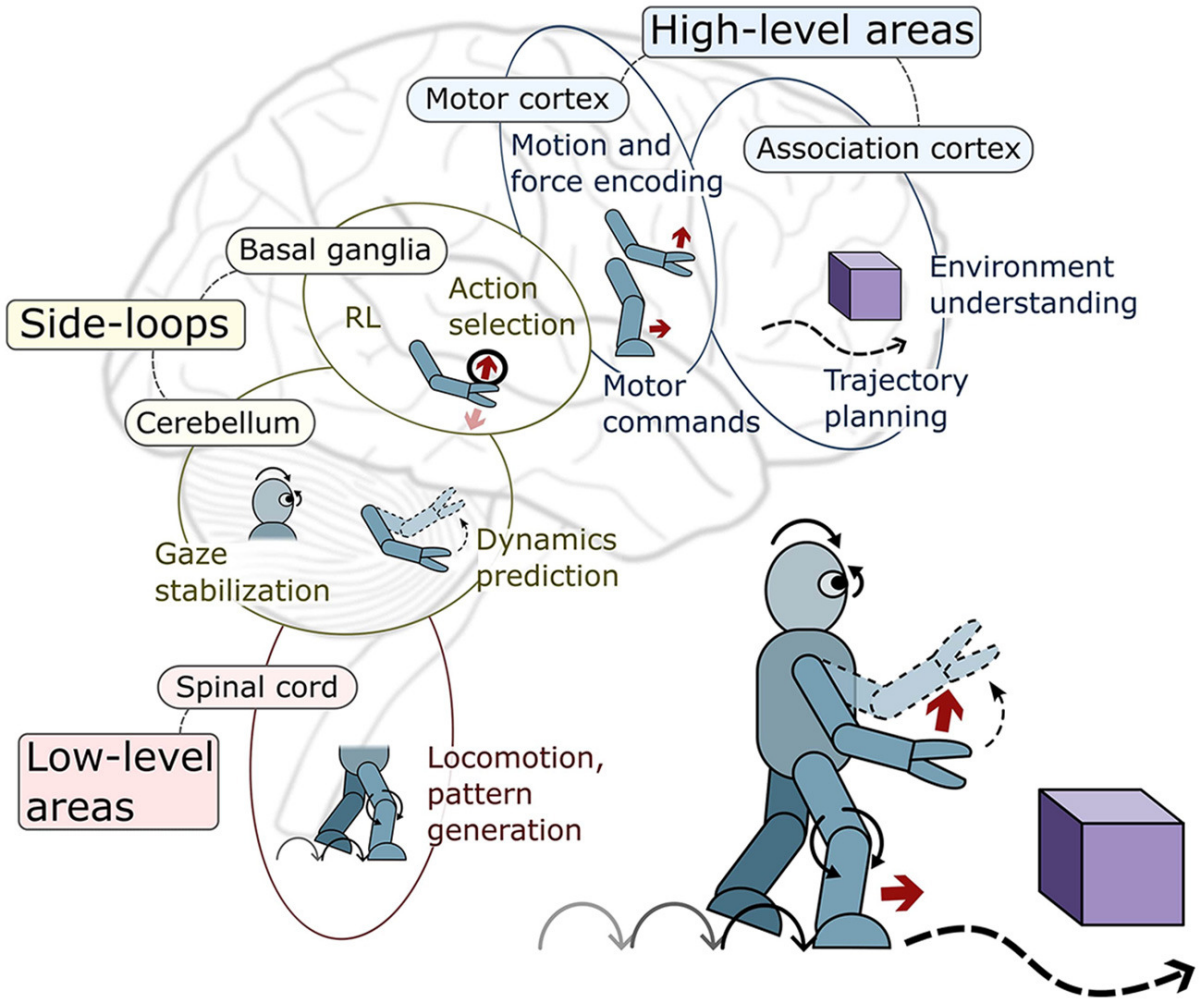
Visual depiction of the brain motor control areas covered in this review and the main robotics control tasks they specialize in.

Since the works on BCs mostly focus on modeling a single or few brain areas, these sections are organized according to the main areas modeled in the brain motor control hierarchy. Inside these sections, the different robotics tasks addressed are presented. This is depicted in Figure 2, and summarized in Table 1. Nevertheless, some works attempt to model a wider range of brain areas and their interconnections; these are known as *systems-level* models.

**Table 1:** Classification of the different works in literature on brain-inspired motor control, sorted by brain areas modeled, robotics tasks addressed, and the approach taken to achieve bio-mimicry (cellular-level or functional).

**Brain motor areas**	**Robotics control tasks**	**Biomimetic modeling approach**	
		**Cellular-level**	**Functional**
High-level areas	Trajectory planning	DeWolf and Eliasmith, 2011; DeWolf et al., 2016; Iacob et al., 2021; Baladron et al., 2023	Gentili et al., 2012, 2016; DeWolf et al., 2023
	Reference conversion, motor commands	DeWolf and Eliasmith, 2011; DeWolf et al., 2016, 2023; Iacob et al., 2021; Zahra et al., 2021a,b, 2022a,b; Baladron et al., 2023	Gentili et al., 2012, 2016; Garrido et al., 2013; Corchado et al., 2019; Abadia et al., 2021a; Zhang et al., 2023
Low-level areas	Locomotion, pattern generation	Pearson et al., 2007, 2010, 2011; Sullivan et al., 2012; Cuevas-Arteaga et al., 2017; Gutierrez-Galan et al., 2020; Polykretis et al., 2020; Spaeth et al., 2020; Strohmer et al., 2020; Antonietti et al., 2022	Massi et al., 2019; Pitchai et al., 2019; Jeppesen et al., 2020; Schmidt et al., 2021; Shao et al., 2022
Basal ganglia	Action selection	Baladron et al., 2023; González-Redondo et al., 2023	Prescott et al., 2006; Mannella and Baldassarre, 2015
	Reinforcement learning	González-Redondo et al., 2023	
Cerebellum	Dynamics learning, disturbance rejection	Carrillo et al., 2008; Luque et al., 2011a,b, 2014; Garrido et al., 2013; Casellato et al., 2014, 2015; Antonietti et al., 2019; Corchado et al., 2019; Naveros et al., 2020; Abadia et al., 2021a,b; Zahra et al., 2021a,b, 2022a	Tolu et al., 2012, 2013, 2020; DeWolf et al., 2016, 2023; Capolei et al., 2019, 2020; Kalidindi et al., 2019; Liu et al., 2020; Wilson et al., 2021; Alepuz et al., 2022; Wilson, 2023; Zhang et al., 2023
	Gaze stabilization	Garrido et al., 2013; Naveros et al., 2020	Wilson et al., 2016

**The brain motor control hierarchy**

The brain's motor system comprises specialized areas dedicated to distinct functions in controlling movement. These regions follow a hierarchical arrangement: higher-level domains oversee broader tasks with considerable abstraction, while lower-level segments focus on individual muscles, delivering precise signals tailored to the task's specifics. Complementing this structure are additional side structures (side loops) responsible for regulating signals within the descending pathways of this hierarchical system. For more details about the brain motor control hierarchy refer to Byrne and Dafny (1997).

- At higher levels, the motor cortex encodes movement force and spatial details, with subdivisions like the premotor and supplementary motor areas handling motion anticipation and kinematic information. Simultaneously, the association cortex aids in environmental representation and action selection based on context.
- At lower levels of the hierarchy, the spinal cord coordinates reflexes, contains Central Pattern Generators (CPGs) for rhythmic movements, controls muscles, and manages vital sensory pathways. Meanwhile, the brainstem acts as a central hub, linking the brain to the body's components, overseeing fundamental functions like balance, breathing, and heart rate.
- Within the side-loop areas, the BG determine suitable motor programs, ensuring the execution of appropriate motor actions. Conversely, the cerebellum contributes to balance, posture, movement coordination, motor learning and the refinement of motor skills.

### 4.1 High-level BC

In the current robotics paradigm, the functions carried out by the motor cortex and the association cortex can be linked to well-studied specific tasks. In the association cortex, this involves trajectory planning and reference generation. Conversely, the tasks relted to the motor cortex encompass spatial and coordinate transformations, along with the creation of inverse dynamics models that convert references into forces or motor commands. Currently, most studies on bio-inspired controllers assume that the references are given and thus exclude the association cortex by replacing it with an analytical trajectory generation module. Moreover, the role of the motor cortex in representing an inverse model is frequently overlooked, often substituted by an analytical controller. This is supplemented by a model within the cerebellum that offers adjustments based on motor or sensory input. Nevertheless, the following works have modeled the functions of the high-level brain motor areas to certain extents.

The authors in DeWolf and Eliasmith (2011) presented a framework for simulating the hierarchical structure of the motor areas, which includes the pre-motor cortex and supplementary motor area to generate high-level reference control signals. However, they did not implement all the areas, and only simulated a model of the motor cortex with SNNs. The system is capable of controlling a simulated 2-DOF arm for a 2-dimensional target reaching task. Some years later, in DeWolf et al. (2016), the same authors introduced the REACH model, a biologically-plausible adaptive hierarchical approach that incorporates the pre-motor cortex. This model generates adaptive dynamical motion primitives to define desired trajectories, controlling a simulated 2-DOF robot arm in tasks such as trajectory tracking and reaching. The primary motor cortex plays a role in learning to model dynamics, supported by the cerebellum. Additionally, it corrects inaccuracies within the robot's Jacobian model. It receives the targets from the pre-motor cortex and the current system state from sensory cortices, and produces low-level signals as motor commands. Iacob et al. (2021) used REACH in a real robot with 3 DOF. They noted that although the tasks are performed successfully, they obtained lower performance than in the original paper. They also proved that the architecture is capable of disturbance rejection. In their work detailed in DeWolf et al. (2023), the REACH model was employed to oversee a 7-DOF simulated robot arm. Notably, the approach did not involve a biomimetic method for generating references. Instead, it was solely utilized to compute joint forces based on desired workspace forces. Throughout these experiments, the authors consistently employed the Neural Engineering Framework (NEF) (Eliasmith and Anderson, 2003), a framework designed for simulating assemblies of spiking neurons.

A functional model of the prefrontal regions was used in Gentili et al. (2012, 2016) to send the desired reaching position. This model learns the relationship between joint and spatial coordinates. In Zahra et al. (2021b), the authors introduced a differential map designed to convert desired velocities within task space into corresponding motor commands within joint space. This mapping is executed through SNNs and is trained offline before task execution. Notably, the same approach is employed by these authors in subsequent works (Zahra et al., 2021a, 2022a,b), where they successfully implemented it to control a real robotic arm. Baladron et al. (2023) used a pre-motor cortex model to generate goal positions, and a motor cortex-basal ganglia loop to select a concrete action. They implemented the algorithm with SNNs to control a simulated arm with 4 DOF. The work discussed in Corchado et al. (2019) implemented an iterative learning controller, specifically a Learning Feedback Controller, designed to simulate the function of the motor cortex. This controller generates control actions by considering the tracking error as a basis for its decision-making process. In Zhang et al. (2023), a recurrent neural network is used as the primary motor cortex that sends motor commands to a musculoskeletal robot, based on several targets to be reached.

Some works use other non-biologically inspired methods but still link them with the cortical areas. For example, in Garrido et al. (2013) and Abadia et al. (2021a), the authors use a conventional trajectory planner with known inverse kinematics and refer to it as the association cortex, and in Garrido et al. (2013), the motor cortex is represented by a recursive Newton-Euler algorithm that provides approximate motor commands given the available inverse dynamic model.

### 4.2 Low-level BC

The most relevant role for robotics carried out by these areas is rhythm generation. This takes place in the spinal cord by groups of neurons within CPGs. The rhythms generated by CPGs can be modulated through sensory feedback to adapt to different scenarios that demand alteration of gait speed or pattern. CPGs have been mostly applied to legged robots, especially hexapod robots.

Several neuromorphic hardware implementations of CPGs have been proposed. In Cuevas-Arteaga et al. (2017), the authors deployed a spiking CPG in SpiNNaker to control an hexapod robot with different gaits that are chosen based on the visual information obtained from an event camera. Gutierrez-Galan et al. (2020) implemented in SpiNNaker a CPG that can change online between three different gaits to control a hexapod. Polykretis et al. (2020) proposed presented a CPG spiking network on Intel^®^Loihi to control an hexapod, showing robustness to noise and different speeds.

Regarding works that take a cellular-level approach in modeling CPGs, Spaeth et al. (2020) presented a minimal network of simulated spiking neurons modulated by sensory feedback that achieves nontrivial behaviors in a flexible walking robot. Strohmer et al. (2020) developed a spiking CPG model capable of continuously changing amplitude, frequency, and phase online, which enables adaptation through feedback.

Massi et al. (2019) modeled several brain motor areas through functional approximations, using non-linear oscillators to model a CPG (functional model). They proposed the use of a learning controller during the optimization process of the locomotion parameters to obtain a final controller configuration with better performance on the walking task of a quadruped robot. In Pitchai et al. (2019), the authors combine a functional CPG with a radial basis function network (RBFN) for locomotion learning of a complex beetle-like robot through reinforcement learning. They focus on the role of the RBFN which determines the shape of the motor patterns, and show that the robot travels faster and is more energy-efficient than using only a CPG. In Shao et al. (2022), the authors control the gait of a gecko-inspired robot by using functional CPGs and a RBFN, combined with exteroceptive sensory feedback to evaluate the terrain. This allows the robot to climb tracks with various slopes and bumps/obstacles, establishing a foundation for climbing robots with adaptive capabilities against rough terrains. Jeppesen et al. (2020) control the oscillations of soft robot through a functional CPG with an adaptation mechanism that modulates the amplitude of the signals upon external perturbations. In Schmidt et al. (2021), the authors compared reflexes, functional CPGs, and a combined approach for controlling a biomimetic robot leg. They found pure reflexes outperformed continuously feedback-adapted CPGs for motion stability and energy efficiency, though pure CPGs allow easier signal modulation. Their results indicate combining reflexes and CPGs could be improved by modulating the control signal shape.

Another application of low-level BC and CPGs in robotics can be found in the control of whisker-like structures for robots, mimicking those found in rodents, to expand the sensory capabilities of mobile robots. In this line of research, Pearson et al. (2007) proposed a multidisciplinary project to reproduce the rodent whisker sensor system in a robotic implementation (*Whiskerbot*). This project replicated the morphology and mechanics of large whiskers, the whiskers movement via a spiking whisker pattern generator (WPG), based on a CPG, and a biologically plausible model of a central nervous system area (specifically the *superior colliculus*) for sensing and controlling the robot behavior with action selection through a basal ganglia model. It effectively demonstrated the adaptation of the whisking pattern after contact, displayed also by rats. The development of this project was continued with (*SCRATCHbot*) (Pearson et al., 2010), where the authors increased the number of whiskers and degrees of freedom to test for more complex WPGs and improving the whisker-environment interaction. In a succeeding work, the same authors developed a new whiskering robot (*Shrewbot*) (Pearson et al., 2011) with improved snout morphology, which allowed to discern between different surface textures by using a statistical classifier on the whiskering sensor data (Sullivan et al., 2012). In simulation, Antonietti et al. (2022) developed a SNN model of the mouse sensorimotor peripheral whisker system, including a CPG, and integrated it into a virtual mouse robot within the Neurorobotics Platform. Together with a cerebellum-inspired controller, they could reproduce active whisking with learning capabilities, matching neural correlates observed in mice.

The research conducted on CPGs for legged-robots, soft-robots and tactile-like (whisker) sensors, has exhibited promising results in terms of both achieving tasks successfully and mirroring the corresponding biological processes. These methods have been effectively deployed in neuromorphic hardware on multiple occasions, showcasing the capacity to replicate the adaptability mechanisms observed in biological systems.

### 4.3 Side-loop BC

#### 4.3.1 Basal ganglia-based controllers

Research on computational models for the BG in robotics primarily revolves around replicating the mechanisms and sub-regions responsible for action selection based on cortical signals (Gurney et al., 2001; Frank, 2011; Véronneau-Veilleux et al., 2021). These models aim to generate specific actions and dynamics while incorporating learning mechanisms through RL. In the context of comprehensive brain-motor system models intended for physical robots operating in a complex environment, BG models are essential. They enable the RL feature essential for learning and selecting optimal high-level actions to operate effectively within the complex environment.

While the BG remain among the least understood brain areas concerning specific connections, interactions, and operational principles, there exists sufficient evidence to model certain functionalities. This evidence allows for testing the distinct roles of their BG sub-regions. The following presented works focused on modeling these roles and functions, employing simulated agents for action selection and robotic or human-like arms for motion-related tasks.

Prescott et al. (2006) integrated a BG model into a small mobile robot, enabling it to select actions amidst various sensory and motivational conditions. While the model effectively selects between competing actions in most cases, it encounters difficulty when faced with two highly probable actions, resulting in oscillation between two behaviors. Mannella and Baldassarre (2015) noted that previous assumptions on how action selection works in the BG are challenged by new perspectives, and proposed a computational model accounting for these. The model was tested on a simulated 3-DOF joint-actuated arm for target reaching tasks, and a 20-DOF hand to reach specific postures. It provided a successful implementation of new properties observed in the BG and not tested before, although they used some biologically implausible simplifications such as supervised learning in certain areas, pointing toward possible further work to bring it closer to biology.

In González-Redondo et al. (2023), the authors proposed a computational model designed to associate complex input patterns with rewarded actions, enabling informed decision-making. A spiking model of the stratium, a BG component, was implemented to perform RL tasks with a simulated agent. This study underscores the pivotal role that different connections and mechanisms within the BG play in facilitating effective action selection. Baladron et al. (2023) adopted a systems-level approach in their implementation, including a cortex-basal ganglia loop. Their system uses novelty-based Hebbian learning to update the interconnections, selecting actions that drive their robotic arm's end-effector to novel positions.

#### 4.3.2 Cerebellum-based controllers

While the previous brain areas contribute significantly to biological motor control and have been modeled to different extents for robotics, the cerebellum distinguishes itself by directly aligning with two pivotal tasks in robotics control: online error correction and dynamics modeling (Albus, 1971; Itō, 1984; Wolpert and Kawato, 1998; Wolpert et al., 1998). The cerebellum is capable of short-term and long-term adaptation (Wulff et al., 2009; Wang et al., 2014). In the short-term adaptation, it swiftly rectifies inaccuracies arising from the motor cortex commands, and effectively rejects disturbances from the external environment. This adaptation facilitates rapid adjustments in motion. In contrast, long-term adaptation in the cerebellum learns detailed dynamic models over time, encompassing the body and environment. This allows for the prediction of sensory and motor outcomes based on the current state and undertaken actions. Consequently, it refines motion beyond the limitations of relying solely on signals from the motor cortex.

The cerebellum has also been suggested to assist in other motion control tasks or even more general learning tasks (Sendhilnathan et al., 2020). Among them, the one that has been mostly studied and tested in humanoid robots with head and eyes is the function of the vestibulo-ocular reflex (VOR) (Troost, 1984), which stabilizes the gaze of the eyes while the head is in motion.

The cerebellum capabilities if reproduced successfully with computational models, may allow novel robotic systems to overcome the issues posed by their nonlinearities and many degrees of freedom, by adapting online to learn the system dynamics or any change in these, as well as to quickly correct any errors that may arise during the motion task due to unexpected disturbances. If successfully replicated through computational models, the capabilities of the cerebellum could potentially empower robots to surmount these challenges.

In Carrillo et al. (2008), authors introduced a real-time spiking model of the cerebellum based on EDLUT (Ros et al., 2006), a SNN simulator developed by their research group. They controlled a 2-DOF simulated and physical robot arm, showing dynamic adaptation to different tasks. This constituted the first real-time application of SNN for robot control. The same research group has developed further work on SNN for robot control based on EDLUT. In Luque et al. (2011a), they showed a cerebellar network that on top of the sensory inputs, it receives additional information regarding the context of the task of a robotic arm. This allows for faster adaptation to newer contexts and for robustness against misleading contextual information. This work was extended in Luque et al. (2011b) by combining a feedforward and a recurrent network topology, which shows robustness against noise. In Luque et al. (2014), they performed further experiments on a simulated robot to show incremental learning of different dynamics models with minimal mutual interference. Garrido et al. (2013) also showed a detailed biomimetic cerebellum architecture that controls a simulated robot arm, and remarked its ability for showing short-term error compensation and long-term adaptation when the model is changed. More recently, Abadia et al. (2021a) have implemented a larger scale SNN based on EDLUT, showing real-time continuous learning on a real compliant robot against unstructured interactions. They also show in Abadia et al. (2021b), that this cerebellar network can deal with non-deterministic delays in robotics applications. Finally, based on the same SNN simulator, Naveros et al. (2020) developed a real time control loop to operate a real robot humanoid performing different VOR tasks.

In an alternate work mentioned in the high-level BC section, DeWolf et al. (2016) and DeWolf et al. (2023) proposed a method that models several brain motor areas using SNNs. However, their focus lies not in replicating the structural properties but rather capturing the functionality of these regions. Notably, their cerebellum model lacks detailed anatomical representations of the microcircuit; instead, it is represented by a generalized SNN. This cerebellar module is designed to learn the inverse dynamics model of the system, targeting the inertia and gravity components within the torque dynamics equation. Simultaneously, it provides corrective control signals to counteract inaccuracies arising from the primary motor cortex module's generated control actions.

Prior works relied on obtaining comprehensive online measurements of robot joint states, essential for the cerebellar model's computation of required torques through inverse dynamics. However, scenarios might arise where only partial joint state information, e.g., joint angles, is available online. In this case, the limitation renders the accuracy of the dynamics model, as it depends on non-measurable states. To tackle this challenge, Zahra et al. (2021b) introduced an innovative solution—an SNN-based differential map function akin to a Jacobian projection. This map relates task-space to joint-space, compensating for incomplete measurements. Their approach involved an optimization procedure to determine the network's hyperparameters, followed by offline training using data gathered from motor babbling. This differential map concept was further explored in Zahra et al. (2021a), where it was combined with a cerebellar network to control a real robot arm. This integration implemented a Smith-predictor (Smith, 1957), a control structure adept ad handling delays, taking inspiration from Tolu et al. (2020). In this structure, the cerebellum acted as a forward-dynamics model, offering task-space sensory adjustments to the differential map. Consequently, the map generated joint commands based on task-space information. Building upon this foundation, the work presented in Zahra et al. (2022a) refined the previous model, introducing a more detailed network and an optimization-driven method to fine-tune the entire network's hyperparameters. This enhanced model showcased its efficacy in controlling a robot arm amidst disturbances, demonstrating significant improvements over prior iterations

Most of the previously presented works adopted a bio-plausible approach, closely emulating the cellular mechanisms found within the cerebellum. However, there is a distinct line of research that diverges from biological plausibility, employing computational models of the cerebellum to achieve remarkable results in robotics tasks. One such noteworthy contribution is the work by Tolu et al. (2012, 2013), where they implemented a modified adaptive filter model (Fujita, 1982; Dean et al., 2010) of the cerebellum. Their approach used the incremental learning algorithm Locally Weighted Projection Regression (LWPR) (Vijayakumar et al., 2005) for long-term dynamics model learning. LWPR is a regression technique that can model non-linear functions in high-dimensional spaces by combining locally linear models that are created and updated online, making it suitable for incremental learning. Additionally, Tolu et al. integrated an extra module for fast adaptation and disturbance rejection, leveraging LWPR's receptive fields (the membership function of each linear model) and updating based on feedback error. This methodology demonstrated successful control of a simulated multi-DOFs robot arm executing cyclic trajectories despite the presence of external disturbances. Continuing this trajectory, Capolei et al. (2019) extended this work by employing a larger cerebellum model to tackle a more complex task: balancing a ball on a table using the arm of a simulated iCub robot. In Capolei et al. (2020), the same researchers introduced an augmented architecture featuring additional synapses and learning rules, aligning more closely with biological principles but still without spiking neuron models. While showcasing good tracking accuracy and robustness against perturbations and noise in controlling 3-DOFs in the simulated iCub robot arm, this model struggles to generalize to all tested scenarios. The authors argued that a more biologically plausible architecture, incorporating additional brain areas could potentially enhance the results. In a similar vein, Tolu et al. (2020) presented a cerebellar-based Smith predictor. Their results highlighted anticipation and adaptation against dynamic changes, demonstrating a significant improvement for tracking accuracy on a real robot arm. The cerebellar model used by Tolu et al. (2012, 2013) was also tested on an underwater robot in simulation, showing the ability to learn a dynamics model with cross-coupling effects and disturbance rejection (Alepuz et al., 2022).

Another work that proposed a functional model of the cerebellum based on echo state networks (ESN) is found in Kalidindi et al. (2019). This model is adept at learning to supplement an approximate inverse kinematics model, specifically in the context of a soft-robot arm engaged in trajectory tracking tasks. In Zhang et al. (2023), the authors use a functional model of the cerebellum and control a musculoskeletal robot. A recurrent neural network acts as the primary motor cortex sending motor commands to the robot based on the targets to be reached. Then, the cerebellar model, which is composed by two networks, first predicts the outcome of these commands with an ESN, and then sends corrective signals with a second network that learns using bio-plausible learning rules. The motion performance greatly improves with the use of the cerebellar model.

An alternate approach to modeling the cerebellum involves implementing the adaptive filter (AF) hypothesis (Fujita, 1982; Dean et al., 2010). In this framework, the cerebellum microcircuit is replicated as a set of second-order low-pass filters with different time constants and multiplicative weights assigned at their outputs. These weights are adapted online from an error signal. The weighted sum of all the filters outputs allows for approximating the system dynamics. Wilson et al. (2016) implemented an AF model of the cerebellum together with an inverse model of the plant represented by a brainstem model, that are used to control a nonlinear electroactive polymer actuator in a VOR application. In Wilson et al. (2021), the same authors have used a similar model of the cerebellum applied to a range of tasks in robot adaptive control and sensorimotor processing. More recently, Wilson (2023) used an AF to control the force of a biomimetic muscle model, by converting the processed AF signals into spiking signals.

Recent evidence suggests a significant role for the cerebellum in supporting RL processes in the brain. Liu et al. (2020) investigated and introduced a cerebellar model that enables RL without relying on explicit teacher signals. This model exhibited success in accomplishing a target-reaching task, demonstrating its effectiveness in both simulated human arm and a real robot arm.

## 5 Conclusion

The works presented in this review on brain-inspired biomimetic control reproduce models of the brain motor areas to different degrees of biological plausibility, conditioned by the current neuroscientific knowledge of these areas.

In some neural areas like the motor cortex and BG, the exact network structure remains unclear for replication in a bio-plausible model. Consequently, functional models are utilized. Conversely, areas like the cerebellum and spinal cord (e.g., CPGs) benefit from a deeper understanding, enabling bio-plausible implementations. Functional models of the motor cortex and BG handle tasks involving high-level information processing, such as planning and generating motor commands based on objectives, and action selection and RL. However, their bio-plausible implementations are limited due to lack of structural knowledge. Furthermore, only simple robotic tasks have been addressed, which currently are easily solved by conventional non-biomimetic methods, like trajectory planners or basic RL. For more advanced tasks (e.g., more complex environments, behaviors and goals), more advanced bio-plausible models of these areas will be necessary. On the contrary, the cerebellum and spinal cord CPGs address fundamental low-level control issues necessary before integrating higher behavioral complexity. Even for basic tasks, accurate execution relies on a robust dynamics model (cerebellum) and adaptive locomotive patterns (CPGs). Hence, currently robotics predominantly focuses on these areas for biologically inspired computational models.

While some works attempt a comprehensive representation of the brain motor system, they often lack complete biological plausibility. Future endeavors should concentrate on modeling multiple areas with bio-plausible frameworks, establishing their interactions (e.g., cerebellum-BG, cerebellum-CPGs) and exploring unexplored brain regions like the brainstem. As robotics evolves to tackle more intricate tasks, holistic models encompassing the entire motor cortex, sub-structures, and their interactions will become imperative.

An important consideration in the development and validation of these biomimetic controllers is the use of real robots vs. simulations. While simulations offer a controlled and safe environment for initial testing and validation with fast iterations, they inevitably fail to capture all the complexities of the real world. On the other hand, physical robots face real-world dynamics with uncertainties and disturbances, providing the ultimate scenario for evaluating the robustness and adaptability of these models. However, working with physical systems introduces additional challenges, such as real-time constraints, sensor noise, and hardware limitations.

Indeed, many of the works presented in this review acknowledge the complex nature of real robotics tasks. Even if testing only in simulation, the introduction of artificial noise and disturbances aims to prove their methods robust and able to generalize to the real world. At the same time, the works that test on real robots demonstrate a higher maturity of their methods and readiness to use in real scenarios. As discussed, the cerebellum plays a big role in noise and disturbance rejection, making it a crucial component for the success of future biomimetic controllers. Ultimately, a combination of simulations and real-robot experiments is likely necessary, with simulations serving as a initial development and testing platform, and real-robot experiments providing the final validation and refinement of these models.

## References

[B1] Abadia I. Naveros F. Garrido J. A. Ros E. Luque N. R. (2021a). On robot compliance: a cerebellar control approach. IEEE Trans. Cybern. 51, 2476–2489. 10.1109/TCYB.2019.2945498 31647453

[B2] Abadia I. Naveros F. Ros E. Carrillo R. R. Luque N. R. (2021b). A cerebellar-based solution to the nondeterministic time delay problem in robotic control. Sci. Robot. 6:eabf2756. 10.1126/scirobotics.abf2756 34516748

[B3] Akopyan F. Sawada J. Cassidy A. Alvarez-Icaza R. Arthur J. Merolla P.. (2015). Truenorth: design and tool flow of a 65 mw 1 million neuron programmable neurosynaptic chip. IEEE Trans. Comput.-Aided Des. Integr. Circuits Syst. 34, 1537–1557. 10.1109/TCAD.2015.2474396

[B4] Albus J. S. (1971). A theory of cerebellar function. Math. Biosci. 10, 25–61. 10.1016/0025-5564(71)90051-4

[B5] Alepuz A. M. Tolu S. Galeazzi R. (2022). Motor learning for manoeuvring control of a remotely operated vehicle. IFAC-PapersOnLine 55, 104–109. 10.1016/j.ifacol.2022.10.416

[B6] Amunts K. Ebell C. Muller J. Telefont M. Knoll A. Lippert T.. (2016). The human brain project: creating a European research infrastructure to decode the human brain. Neuron 92, 574–581. 10.1016/j.neuron.2016.10.046 27809997

[B7] Antonietti A. Geminiani A. Negri E. D'Angelo E. Casellato C. Pedrocchi A.. (2022). Brain-inspired spiking neural network controller for a neurorobotic whisker system. Front. Neurorobot. 16:817948. 10.3389/fnbot.2022.817948 35770277 PMC9234954

[B8] Antonietti A. Martina D. Casellato C. D'Angelo E. Pedrocchi A. (2019). Control of a humanoid nao robot by an adaptive bioinspired cerebellar module in 3D motion tasks. Comput. Intell. Neurosci. 2019:4862157. 10.1155/2019/4862157 30833964 PMC6369512

[B9] Asano Y. Okada K. Inaba M. (2017). Design principles of a human mimetic humanoid: humanoid platform to study human intelligence and internal body system. Sci. Rob. 2:eaaq0899. 10.1126/scirobotics.aaq0899 33157878

[B10] Baladron J. Vitay J. Fietzek T. Hamker F. H. (2023). The contribution of the basal ganglia and cerebellum to motor learning: a neuro-computational approach. PLoS Comput. Biol. 19, 1–29. 10.1371/journal.pcbi.1011243 37011086 PMC10101648

[B11] Berberich J. Kohler J. Muller M. A. Allgower F. (2021). Data-driven model predictive control with stability and robustness guarantees. IEEE Trans. Automat. Contr. 66., 1702–1717 10.1109/TAC.2020.3000182

[B12] Bhattacharya D. Cheng L. K. Xu W. (2020). Sparse machine learning discovery of dynamic differential equation of an esophageal swallowing robot. IEEE Trans. Ind. Electron. 67, 4711–4720. 10.1109/TIE.2019.2928239

[B13] Brunton S. L. Proctor J. L. Kutz J. N. (2016). Discovering governing equations from data by sparse identification of nonlinear dynamical systems. Proc. Natl. Acad. Sci. USA. 113, 3932–3937. 10.1073/pnas.1517384113 27035946 PMC4839439

[B14] Byrne J. H. Dafny N. (1997). Neuroscience Online: An Electronic Textbook for the Neurosciences. Department of Neurobiology and Anatomy; The University of Texas Medical School at Houston.

[B15] Capolei M. C. Andersen N. A. Lund H. H. Falotico E. Tolu S. (2020). A cerebellar internal models control architecture for online sensorimotor adaptation of a humanoid robot acting in a dynamic environment. IEEE Robot. Autom. Lett. 5, 80–87. 10.1109/LRA.2019.2943818

[B16] Capolei M. C. Angelidis E. Falotico E. Lund H. H. Tolu S. (2019). A biomimetic control method increases the adaptability of a humanoid robot acting in a dynamic environment. Front. Neurorobot. 13:70. 10.3389/fnbot.2019.00070 31555117 PMC6722230

[B17] Carpi F. (2016). Electromechanically Active Polymers: A Concise Reference. New York, NY: Springer. 10.1007/978-3-319-31767-0

[B18] Carrillo R. R. Ros E. Boucheny C. Coenen O. J. (2008). A real-time spiking cerebellum model for learning robot control. BioSystems 94, 18–27. 10.1016/j.biosystems.2008.05.008 18616974

[B19] Casellato C. Antonietti A. Garrido J. A. Carrillo R. R. Luque N. R. Ros E.. (2014). Adaptive robotic control driven by a versatile spiking cerebellar network. PLoS ONE 9:e0112265. 10.1371/journal.pone.0112265 25390365 PMC4229206

[B20] Casellato C. Antonietti A. Garrido J. A. Ferrigno G. D'Angelo E. Pedrocchi A.. (2015). Distributed cerebellar plasticity implements generalized multiple-scale memory components in real-robot sensorimotor tasks. Front. Comput. Neurosci. 9:24. 10.3389/fncom.2015.00024 25762922 PMC4340181

[B21] Chen J. Zhang M. Yang Z. Xia L. (2021). “A robust data-driven approach for dynamics model identification in trajectory planning,” in 2021 IEEE/RSJ International Conference on Intelligent Robots and Systems (IROS) (Prague: IEEE). 10.1109/IROS51168.2021.9635979

[B22] Cianchetti M. Laschi C. Menciassi A. Dario P. (2018). Biomedical applications of soft robotics. 10.1038/s41578-018-0022-y

[B23] Coelho J. Ribeiro F. Dias B. Lopes G. Flores P. (2021). Trends in the control of hexapod robots: a survey. Robotics, 10. 10.3390/robotics10030100

[B24] Corchado C. Antonietti A. Capolei M. C. Casellato C. Tolu S. (2019). “Integration of paired spiking cerebellar models for voluntary movement adaptation in a closed-loop neuro-robotic experiment. A simulation study,” in 2019 IEEE International Conference on Cyborg and Bionic Systems (CBS) (Munich: IEEE). 10.1109/CBS46900.2019.9114412

[B25] Cuevas-Arteaga B. Dominguez-Morales J. P. Rostro-Gonzalez H. Espinal A. Jimenez-Fernandez A. F. Gomez-Rodriguez F.. (2017). “A spinnaker application: design, implementation and validation of SCPGS,” in Lecture Notes in Computer Science (including subseries Lecture Notes in Artificial Intelligence and Lecture Notes in Bioinformatics), Vol. 10305 (Cham: Springer). 10.1007/978-3-319-59153-7_47

[B26] Davies M. Srinivasa N. Lin T. H. Chinya G. Cao Y. Choday S. H.. (2018). Loihi: a neuromorphic manycore processor with on-chip learning. IEEE Micro 38, 82–89. 10.1109/MM.2018.112130359

[B27] De Santos P. G. Garcia E. Estremera J. (2006). Quadrupedal Locomotion: An Introduction to the Control of Four-Legged Robots, Vol. 1. London: Springer.

[B28] Dean P. Porrill J. Ekerot C. F. Jörntell H. (2010). The cerebellar microcircuit as an adaptive filter: experimental and computational evidence. Nat. Rev. Neurosci. 11, 30–43. 10.1038/nrn2756 19997115

[B29] DeWolf T. (2021). Spiking neural networks take control. Sci. Robot. 6:eabk3268. 10.1126/scirobotics.abk3268 34516751

[B30] DeWolf T. Eliasmith C. (2011). The neural optimal control hierarchy for motor control. J. Neural. Eng. 8:065009. 10.1088/1741-2560/8/6/065009 22056418

[B31] DeWolf T. Patel K. Jaworski P. Leontie R. Hays J. Eliasmith C.. (2023). Neuromorphic control of a simulated 7-dof arm using loihi. Neuromorph. Comput. Eng. 3:014007. 10.1088/2634-4386/acb286

[B32] DeWolf T. Stewart T. C. Slotine J. J. Eliasmith C. (2016). A spiking neural model of adaptive arm control. Proc. R. Soc. B Biol. Sci. 283:20162134. 10.1098/rspb.2016.2134 27903878 PMC5136600

[B33] Diamond A. Knight R. Devereux D. Holland O. (2012). Anthropomimetic robots: concept, construction and modelling. Int. J. Adv. Robot. Syst. 9:209. 10.5772/52421

[B34] Dong Y. Zhao D. Li Y. Zeng Y. (2023). An unsupervised stdp-based spiking neural network inspired by biologically plausible learning rules and connections. Neural Netw. 165, 799–808. 10.1016/j.neunet.2023.06.019 37418862

[B35] Eliasmith C. Anderson C. (2003). Neural Engineering: Computation, Representation and Dynamics in Neurobiological Systems. Cambridge: MIT Press.

[B36] Fasel U. Kaiser E. Kutz J. N. Brunton B. W. Brunton S. L. (2021). “Sindy with control: a tutorial,” in 2021 60th IEEE Conference on Decision and Control (CDC) (Austin, TX: IEEE). 10.1109/CDC45484.2021.9683120

[B37] Feldman D. E. (2012). The spike-timing dependence of plasticity. Neuron 75, 556–571. 10.1016/j.neuron.2012.08.001 22920249 PMC3431193

[B38] Fliess M. Join C. (2013). Model-free control. Int. J. Control 86, 2228–2252. 10.1080/00207179.2013.810345

[B39] Frank M. J. (2011). Computational models of motivated action selection in corticostriatal circuits. Curr. Opin. Neurobiol. 21, 381–386. 10.1016/j.conb.2011.02.013 21498067

[B40] Fujita M. (1982). Adaptive filter model of the cerebellum. Biol. Cybern. 45, 195–206. 10.1007/BF00336192 7171642

[B41] Furber S. B. Galluppi F. Temple S. Plana L. A. (2014). The spinnaker project. Proc. IEEE 102, 652–655. 10.1109/JPROC.2014.2304638

[B42] Garrido J. A. Luque N. R. D'Angelo E. Ros E. (2013). Distributed cerebellar plasticity implements adaptable gain control in a manipulation task: a closed-loop robotic simulation. Front. Neural Circuits 7:159. 10.3389/fncir.2013.00159 24130518 PMC3793577

[B43] Gentili R. J. Oh H. Kregling A. V. Reggia J. A. (2016). A cortically-inspired model for inverse kinematics computation of a humanoid finger with mechanically coupled joints. Bioinspir. Biomime. 11:036013. 10.1088/1748-3190/11/3/036013 27194213

[B44] Gentili R. J. Oh H. Molina J. Reggia J. A. Contreras-Vidal J. L. (2012). “Cortex inspired model for inverse kinematics computation for a humanoid robotic finger,” in 2012 Annual International Conference of the IEEE Engineering in Medicine and Biology Society (San Diego, CA: IEEE). 10.1109/EMBC.2012.6346608 23366569 PMC3694134

[B45] Ghosh-Dastidar S. Adeli H. (2009). Spiking neural networks. Int. J. Neural Syst. 19, 295–308. 10.1142/S0129065709002002 19731402

[B46] González-Redondo Á. Garrido J. Arrabal F. N. Kotaleski J. H. Grillner S. Ros E. (2023). Reinforcement learning in a spiking neural model of striatum plasticity. Neurocomputing 548:126377. 10.1016/j.neucom.2023.126377

[B47] Gurney K. Prescott T. J. Redgrave P. (2001). A computational model of action selection in the basal ganglia. I. A new functional anatomy. Biol. Cybern. 84, 401–410. 10.1007/PL00007984 11417052

[B48] Gutierrez-Galan D. Dominguez-Morales J. P. Perez-Peña F. Jimenez-Fernandez A. Linares-Barranco A. (2020). Neuropod: a real-time neuromorphic spiking CPG applied to robotics. Neurocomputing 381, 10–19. 10.1016/j.neucom.2019.11.007

[B49] Hao Y. Huang X. Dong M. Xu B. (2020). A biologically plausible supervised learning method for spiking neural networks using the symmetric stdp rule. Neural Netw. 121, 387–395. 10.1016/j.neunet.2019.09.007 31593843

[B50] Hoi S. C. Sahoo D. Lu J. Zhao P. (2021). Online learning: a comprehensive survey. Neurocomputing 459, 249–289. 10.1016/j.neucom.2021.04.112

[B51] Iacob S. Kwisthout J. Thill S. (2021). “From models of cognition to robot control and back using spiking neural networks,” in Biomimetic and Biohybrid Systems. Living Machines 2020. Lecture Notes in Computer Science, Vol. 12413, eds. V. Vouloutsi, A. Mura, F. Tauber, T. Speck, T. J. Prescott, and P. F. M. J. Verschure (Chma: Springer), 176–191. 10.1007/978-3-030-64313-3_18

[B52] Itō M. (1984). The Cerebellum and Neural Control. New York, NY: Raven Press.

[B53] Jeppesen M. H. Jørgensen J. Manoonpong P. (2020). “Adaptive neural cpg-based control for a soft robotic tentacle,” in Neural Information Processing. ICONIP 2020. Lecture Notes in Computer Science, Vol 12533, eds. H. Yang, K. Pasupa, A. C. S. Leung, J. T. Kwok, J. H. Chan, and I. King (Cham: Springer), 762–774. 10.1007/978-3-030-63833-7_64

[B54] Kalidindi H. T. Thuruthel T. G. Laschi C. Falotico E. (2019). “Cerebellum-inspired approach for adaptive kinematic control of soft robots,” in 2019 2nd IEEE International Conference on Soft Robotics (RoboSoft) (Seoul: IEEE). 10.1109/ROBOSOFT.2019.8722735

[B55] Kim Y. Li Y. Park H. Venkatesha Y. Hambitzer A. Panda P.. (2023). Exploring temporal information dynamics in spiking neural networks. Proc. AAAI Conf. Artif. Intell. 37, 8308–8316. 10.1609/aaai.v37i7.26002

[B56] Kirkpatrick J. Pascanu R. Rabinowitz N. Veness J. Desjardins G. Rusu A. A.. (2017). Overcoming catastrophic forgetting in neural networks. Proc. Natl. Acad. Sci. USA. 114, 3521–3526. 10.1073/pnas.1611835114 28292907 PMC5380101

[B57] Liu R. Zhang Q. Chen Y. Wang J. Yang L. (2020). A biologically constrained cerebellar model with reinforcement learning for robotic limb control. IEEE Access 8, 2169–3536. 10.1109/ACCESS.2020.3042994

[B58] Luque N. R. Carrillo R. R. Naveros F. Garrido J. A. Sáez-Lara M. J. (2014). Integrated neural and robotic simulations. Simulation of cerebellar neurobiological substrate for an object-oriented dynamic model abstraction process. Rob. Auton. Syst. 62, 1702–1716. 10.1016/j.robot.2014.08.002

[B59] Luque N. R. Garrido J. A. Carrillo R. R. Coenen O. J. Ros E. (2011a). Cerebellar input configuration toward object model abstraction in manipulation tasks. IEEE Trans. Neural Netw. 22, 1321–1328. 10.1109/TNN.2011.2156809 21708499

[B60] Luque N. R. Garrido J. A. Carrillo R. R. Tolu S. Ros E. (2011b). Adaptive cerebellar spiking model embedded in the control loop: context switching and robustness against noise. Int. J. Neural Syst. 21, 385–401. 10.1142/S0129065711002900 21956931

[B61] Lyashenko V. Ahmad M. A. Belova N. Sotnik S. (2021). Modern walking robots: a brief overview. Int. J. Recent Technol. Appl. Sci. 3, 32–39. 10.36079/lamintang.ijortas-0302.252

[B62] Maass W. (1997). Networks of spiking neurons: the third generation of neural network models. Neural Netw. 10, 1659–1671. 10.1016/S0893-6080(97)00011-7

[B63] Mannella F. Baldassarre G. (2015). Selection of cortical dynamics for motor behaviour by the basal ganglia. Biol. Cybern. 109, 575–595. 10.1007/s00422-015-0662-6 26537483 PMC4656718

[B64] Massi E. Vannucci L. Albanese U. Capolei M. C. Vandesompele A. Urbain G.. (2019). Combining evolutionary and adaptive control strategies for quadruped robotic locomotion. Front. Neurorobot. 13:71. 10.3389/fnbot.2019.00071 31555118 PMC6727738

[B65] Mirvakili S. M. Hunter I. W. (2018). Artificial muscles: Mechanisms, applications, and challenges. Adv. Mater. 30:1704407. 10.1002/adma.201704407 29250838

[B66] Naveros F. Luque N. R. Ros E. Arleo A. (2020). Vor adaptation on a humanoid icub robot using a spiking cerebellar model. IEEE Trans. Cybern. 50. 10.1109/TCYB.2019.2899246 30835236

[B67] Nguyen-Tuong D. Peters J. Seeger M. (2009). “Local Gaussian process regression for real time online model learning and control,” in 2008 IEEE/RSJ International Conference on Intelligent Robots and Systems (Nice: IEEE). 10.1109/IROS.2008.4650850

[B68] Pearson M. J. Mitchinson B. Sullivan J. C. Pipe A. G. Prescott T. J. (2011). Biomimetic vibrissal sensing for robots. R. Soc. Philos. Trans. B 366, 3085–3096. 10.1098/rstb.2011.0164 21969690 PMC3172604

[B69] Pearson M. J. Mitchinson B. Welsby J. Pipe T. Prescott T. J. (2010). “Scratchbot: active tactile sensing in a whiskered mobile robot,” in From Animals to Animats 11. SAB 2010. Lecture Notes in Computer Science, Vol. 6226, eds. S. Doncieux, B. Girard, A. Guillot, J. Hallam, J. A. Meyer, and J. B. Mouret (Berlin: Springer), 93–103. 10.1007/978-3-642-15193-4_9

[B70] Pearson M. J. Pipe A. G. Melhuish C. Mitchinson B. Prescott T. J. (2007). Whiskerbot: a robotic active touch system modeled on the rat whisker sensory system. Adapt. Behav. 15, 223–240. 10.1177/1059712307082089

[B71] Pehle C. Billaudelle S. Cramer B. Kaiser J. Schreiber K. Stradmann Y.. (2022). The brainscales-2 accelerated neuromorphic system with hybrid plasticity. Front. Neurosci. 16:795876. 10.3389/fnins.2022.795876 35281488 PMC8907969

[B72] Pezzato C. Ferrari R. Corbato C. H. (2020). A novel adaptive controller for robot manipulators based on active inference. IEEE Robot. Autom. Lett. 5, 2973–2980. 10.1109/LRA.2020.2974451

[B73] Pietrzak P. Szczȩsny S. Huderek D. Przyborowski Ł. (2023). Overview of spiking neural network learning approaches and their computational complexities. Sensors 23:3037. 10.3390/s23063037 36991750 PMC10053242

[B74] Pitchai M. Xiong X. Thor M. Billeschou P. Mailänder P. L. Leung B.. (2019). “CPG driven rbf network control with reinforcement learning for gait optimization of a dung beetle-like robot,” in Artificial Neural Networks and Machine Learning – ICANN 2019: Theoretical Neural Computation. ICANN 2019. Lecture Notes in Computer Science, Vol. 11727, eds. I. Tetko, V. Kurkova, P. Karpov, and F. Theis (Cham: Springer), 698–710. 10.1007/978-3-030-30487-4_53

[B75] Polydoros A. S. Nalpantidis L. (2017). Survey of model-based reinforcement learning: applications on robotics. J. Intell. Robot. Syst. Theory Appl. 86, 153–173. 10.1007/s10846-017-0468-y

[B76] Polykretis I. Tang G. Michmizos K. P. (2020). “An astrocyte-modulated neuromorphic central pattern generator for hexapod robot locomotion on Intel's Loihi,” in ICONS 2020: International Conference on Neuromorphic Systems 2020 (New York, NY: ACM), 1–9. 10.1145/3407197.3407205

[B77] Prescott T. J. González F. M. M. Gurney K. Humphries M. D. Redgrave P. (2006). A robot model of the basal ganglia: behavior and intrinsic processing. Neural Netw. 19, 31–61. 10.1016/j.neunet.2005.06.049 16153803

[B78] Qiao G. C. Hu S. G. Wang J. J. Zhang C. M. Chen T. P. Ning N.. (2019). A neuromorphic-hardware oriented bio-plausible online-learning spiking neural network model. IEEE Access 7, 2169–3536. 10.1109/ACCESS.2019.2919163

[B79] Qiao H. Wu Y. X. Zhong S. L. Yin P. J. Chen J. H. (2023). Brain-inspired intelligent robotics: theoretical analysis and systematic application. Mach. Intell. Res. 20, 1–18. 10.1007/s11633-022-1390-8

[B80] Rathi N. Chakraborty I. Kosta A. Sengupta A. Ankit A. Panda P.. (2023). Exploring neuromorphic computing based on spiking neural networks: algorithms to hardware. ACM Comput. Surveys 55, 1–23. 10.1145/3571155

[B81] Reinhart R. F. Shareef Z. Steil J. J. (2017). Hybrid analytical and data-driven modeling for feed-forward robot control. Sensors 17:311. 10.3390/s17020311 28208697 PMC5336126

[B82] Ros E. Carrillo R. Ortigosa E. M. Barbour B. Agís R. (2006). Event-driven simulation scheme for spiking neural networks using lookup tables to characterize neuronal dynamics. Neural Comput. 18, 2959–2993. 10.1162/neco.2006.18.12.2959 17052155

[B83] Santina C. D. Duriez C. Rus D. (2023). Model-based control of soft robots: a survey of the state of the art and open challenges. IEEE Control Syst. 43, 30–65. 10.1109/MCS.2023.3253419

[B84] Schmidt A. Feldotto B. Gumpert T. Seidel D. Albu-Schäffer A. Stratmann P.. (2021). Adapting highly-dynamic compliant movements to changing environments: a benchmark comparison of reflex- vs. cpg-based control strategies. Front. Neurorobot. 15:762431. 10.3389/fnbot.2021.762431 34955801 PMC8709475

[B85] Sendhilnathan N. Ipata A. E. Goldberg M. E. (2020). Neural correlates of reinforcement learning in mid-lateral cerebellum. Neuron 106, 188–198.e5. 10.1016/j.neuron.2019.12.032 32001108 PMC8015782

[B86] Shao D. Wang Z. Ji A. Dai Z. Manoonpong P. (2022). A gecko-inspired robot with cpg-based neural control for locomotion and body height adaptation. Bioinspir. Biomim 17:036008. 10.1088/1748-3190/ac5a3c 35236786

[B87] Singh B. Kumar R. Singh V. P. (2022). Reinforcement learning in robotic applications: a comprehensive survey. Artif. Intell. Rev. 55, 945–990. 10.1007/s10462-021-09997-9

[B88] Smith O. J. (1957). Closer control of loops with dead time. Chem. Eng. Prog. 53, 217–219.

[B89] Spaeth A. Tebyani M. Haussler D. Teodorescu M. (2020). “Neuromorphic closed-loop control of a flexible modular robot by a simulated spiking central pattern generator,” in 2020 3rd IEEE International Conference on Soft Robotics (RoboSoft) (New Haven, CT: IEEE). 10.1109/RoboSoft48309.2020.9116007

[B90] Strohmer B. Manoonpong P. Larsen L. B. (2020). Flexible spiking CPGS for online manipulation during hexapod walking. Front. Neurorobot. 14:41. 10.3389/fnbot.2020.00041 32676022 PMC7333644

[B91] Sullivan J. C. Mitchinson B. Pearson M. J. Evans M. Lepora N. F. Fox C. W.. (2012). Tactile discrimination using active whisker sensors. IEEE Sens. J. 12, 350–362. 10.1109/JSEN.2011.2148114

[B92] Taherkhani A. Belatreche A. Li Y. Cosma G. Maguire L. P. McGinnity T. M.. (2020). A review of learning in biologically plausible spiking neural networks. Neural Netw. 122, 253–272. 10.1016/j.neunet.2019.09.036 31726331

[B93] Tang Z. Wang P. Xin W. Laschi C. (2022). Learning-based approach for a soft assistive robotic arm to achieve simultaneous position and force control. IEEE Robot. Automat. Lett. 7, 8315–8322. 10.1109/LRA.2022.3185786

[B94] Tavanaei A. Ghodrati M. Kheradpisheh S. R. Masquelier T. Maida A. (2019). Deep learning in spiking neural networks. Neural Netw. 111, 47–63. 10.1016/j.neunet.2018.12.002 30682710

[B95] Tolu S. Capolei M. C. Vannucci L. Laschi C. Falotico E. Hernández M. V.. (2020). A cerebellum-inspired learning approach for adaptive and anticipatory control. Int. J. Neural Syst. 30. 10.1142/S012906571950028X 31771377

[B96] Tolu S. Strohmer B. Zahra O. (2023). Perspective on investigation of neurodegenerative diseases with neurorobotics approaches. Neuromorphic Comput. Eng. 3. 10.1088/2634-4386/acc2e1

[B97] Tolu S. Vanegas M. Garrido J. A. Luque N. R. Ros E. (2013). Adaptive and predictive control of a simulated robot arm. Int. J. Neural Syst. 23:1350010. 10.1142/S012906571350010X 23627657

[B98] Tolu S. Vanegas M. Luque N. R. Garrido J. A. Ros E. (2012). Bio-inspired adaptive feedback error learning architecture for motor control. Biol. Cybern. 106, 507–522. 10.1007/s00422-012-0515-5 22907270

[B99] Troost B. T. (1984). The neurology of eye movements. Neurology 34:845. 10.1212/WNL.34.6.845-c

[B100] Véronneau-Veilleux F. Robaey P. Ursino M. Nekka F. (2021). An integrative model of Parkinson's disease treatment including levodopa pharmacokinetics, dopamine kinetics, basal ganglia neurotransmission and motor action throughout disease progression. J. Pharmacokinet. Pharmacodyn. 48, 133–148. 10.1007/s10928-020-09723-y 33084988

[B101] Vijayakumar S. D'Souza A. Schaal S. (2005). LWPR: a scalable method for incremental online learning in high dimensions. Neural Comput. 17, 2602–2634. 10.1162/089976605774320557 16212764

[B102] Walker J. Zidek T. Harbel C. Yoon S. Strickland F. S. Kumar S.. (2020). Soft robotics: a review of recent developments of pneumatic soft actuators. Actuators 9:3. 10.3390/act9010003

[B103] Wang W. Nakadate K. Masugi-Tokita M. Shutoh F. Aziz W. Tarusawa E.. (2014). Distinct cerebellar engrams in short-term and long-term motor learning. Proc. Natl. Acad. Sci. USA. 111, E188–E193. 10.1073/pnas.1315541111 24367085 PMC3890858

[B104] Wang X. Li Y. Kwok K. W. (2021). A survey for machine learning-based control of continuum robots. Front. Robot. AI 8:730330. 10.3389/frobt.2021.730330 34692777 PMC8527450

[B105] Wang Y. Gao F. Doyle F. J. (2009). Survey on iterative learning control, repetitive control, and run-to-run control. J. Process Control 19, 1589–1600. 10.1016/j.jprocont.2009.09.006

[B106] Westervelt E. R. Grizzle J. W. Chevallereau C. Choi J. H. Morris B. (2018). Feedback Control of Dynamic Bipedal Robot Locomotion. CRC Press.

[B107] Wilson E. (2023). Adaptive filter model of cerebellum for biological muscle control with spike train inputs. Neural Comput. 35, 1938–1969. 10.1162/neco_a_01617 37844325

[B108] Wilson E. D. Assaf T. Pearson M. J. Rossiter J. M. Anderson S. R. Porrill J.. (2016). Cerebellar-inspired algorithm for adaptive control of nonlinear dielectric elastomerbased artificial muscle. J. R. Soc. Interface 13:20160547. 10.1098/rsif.2016.0547 27655667 PMC5046955

[B109] Wilson E. D. Assaf T. Rossiter J. M. Dean P. Porrill J. Anderson S. R.. (2021). A multizone cerebellar chip for bioinspired adaptive robot control and sensorimotor processing: a multizone cerebellar chip for bioinspired adaptive robot control and sensorimotor processing. J. R. Soc. Interface 18:20200750. 10.1098/rsif.2020.0750 33499769 PMC7879753

[B110] Wolpert D. M. Kawato M. (1998). Multiple paired forward and inverse models for motor control. Neural Netw. 11, 1317–1329. 10.1016/S0893-6080(98)00066-5 12662752

[B111] Wolpert D. M. Miall R. C. Kawato M. (1998). Internal models in the cerebellum. Trends Cogn. Sci. 2, 338–347. 10.1016/S1364-6613(98)01221-2 21227230

[B112] Wulff P. Schonewille M. Renzi M. Viltono L. Sassoè-Pognetto M. Badura A.. (2009). Synaptic inhibition of purkinje cells mediates consolidation of vestibulo-cerebellar motor learning. Nat. Neurosci. 12, 1042–1049. 10.1038/nn.2348 19578381 PMC2718327

[B113] Yamazaki K. Vo-Ho V. K. Bulsara D. Le N. (2022). *Spiking neural networks and their* applications: a review. Brain Sci. 12:863. 10.3390/brainsci12070863 35884670 PMC9313413

[B114] Yang S. Chen B. (2023a). Effective surrogate gradient learning with high-order information bottleneck for spike-based machine intelligence. IEEE Trans. Neural Netw. Learn. Syst. 10.1109/TNNLS.2023.3329525 37991917

[B115] Yang S. Chen B. (2023b). Snib: improving spike-based machine learning using nonlinear information bottleneck. IEEE Trans. Syst. Man Cybern. Syst. 53, 7852–7863. 10.1109/TSMC.2023.3300318

[B116] Yang S. Pang Y. Wang H. Lei T. Pan J. Wang J.. (2023a). Spike-driven multi-scale learning with hybrid mechanisms of spiking dendrites. Neurocomputing 542:126240. 10.1016/j.neucom.2023.126240

[B117] Yang S. Wang H. Chen B. (2023b). Sibols: robust and energy-efficient learning for spike-based machine intelligence in information bottleneck framework. IEEE Trans. Cogn. Dev. Syst. 1–13. 10.1109/TCDS.2023.3329532

[B118] Yang S. Wang H. Pang Y. Azghadi M. R. Linares-Barranco B. (2024a). Nadol: neuromorphic architecture for spike-driven online learning by dendrites. IEEE Trans. Biomed. Circuits Syst. 18, 186–199. 10.1109/TBCAS.2023.3316968 37725735

[B119] Yang S. Wang H. Pang Y. Jin Y. Linares-Barranco B. (2024b). Integrating visual perception with decision making in neuromorphic fault-tolerant quadruplet-spike learning framework. IEEE Trans. Sys. Man Cybern. Syst. 54, 1502–1514. 10.1109/TSMC.2023.3327142

[B120] Yang S. Wang J. Deng B. Azghadi M. R. Linares-Barranco B. (2022a). Neuromorphic context-dependent learning framework with fault-tolerant spike routing. IEEE Tran. Neural Netw. Learn. Syst. 33, 7126–7140. 10.1109/TNNLS.2021.3084250 34115596

[B121] Yang S. Wang J. Hao X. Li H. Wei X. Deng B.. (2022b). Bicoss: toward large-scale cognition brain with multigranular neuromorphic architecture. IEEE Trans. Neural Netw. Learn. Syst. 33, 2801–2815. 10.1109/TNNLS.2020.3045492 33428574

[B122] Yang S. Wang J. Zhang N. Deng B. Pang Y. Azghadi M. R.. (2021). Cerebellumorphic: large-scale neuromorphic model and architecture for supervised motor learning. IEEE Trans. Neural Netw. Learn. Syst. 33, 4398–4412. 10.1109/TNNLS.2021.3057070 33621181

[B123] Yao M. Zhao G. Zhang H. Hu Y. Deng L. Tian Y.. (2023). Attention spiking neural networks. IEEE Trans. Pattern Anal. Mach. Intell. 45, 9393–9410. 10.1109/TPAMI.2023.3241201 37022261

[B124] Young A. R. Dean M. Plank J. S. Rose G. S. (2019). A review of spiking neuromorphic hardware communication systems. IEEE Access 7, 135606–135620. 10.1109/ACCESS.2019.2941772

[B125] Zahra O. Navarro-Alarcon D. Tolu S. (2021a). “A fully spiking neural control system based on cerebellar predictive learning for sensor-guided robots,” in 2021 IEEE International Conference on Robotics and Automation (ICRA). (New York, NY: ACM). 10.1109/ICRA48506.2021.9561127

[B126] Zahra O. Navarro-Alarcon D. Tolu S. (2022a). A neurorobotic embodiment for exploring the dynamical interactions of a spiking cerebellar model and a robot arm during vision-based manipulation tasks. Int. J. Neural Syst. 32:2150028. 10.1142/S0129065721500283 34003083

[B127] Zahra O. Tolu S. Navarro-Alarcon D. (2021b). Differential mapping spiking neural network for sensor-based robot control. Bioinspir. Biomim. 16:036008. 10.1088/1748-3190/abedce 33706302

[B128] Zahra O. Tolu S. Zhou P. Duan A. Navarro-Alarcon D. (2022b). A bio-inspired mechanism for learning robot motion from mirrored human demonstrations. Front. Neurorobot. 16:826410. 10.3389/fnbot.2022.826410 35360830 PMC8963868

[B129] Zhang J. Chen J. Wu W. Qiao H. (2023). A cerebellum-inspired prediction and correction model for motion control of a musculoskeletal robot. IEEE Trans. Cogn. Dev. Syst. 15, 1209–1223. 10.1109/TCDS.2022.3200839

[B130] Zhang T. Mo H. (2021). Reinforcement learning for robot research: a comprehensive review and open issues. Int. J. Adv. Robot. Syst. 18. 10.1177/17298814211007305

